# Fourteen weeks of multicomponent training associated with flexibility training modifies postural alignment, joint range of motion and modulates blood pressure in physically inactive older women: a randomized clinical trial

**DOI:** 10.3389/fphys.2023.1172780

**Published:** 2023-11-02

**Authors:** Andressa C. S. Sobrinho, Cicero Jonas R. Benjamim, Mariana Luciano de Almeida, Guilherme da Silva Rodrigues, Laryssa Grazielle Feitosa Lopes, João Gabriel Ribeiro de Lima, Carlos Roberto Bueno Júnior

**Affiliations:** ^1^ Ribeirão Preto Medical School, University of São Paulo (USP), São Paulo, Brazil; ^2^ College of Nursing of Ribeirão Preto, University of São Paulo (USP), São Paulo, Brazil; ^3^ School of Physical Education and Sport of Ribeirão Preto, University of São Paulo (USP), São Paulo, Brazil

**Keywords:** muscle compensation, healthcare, range of motion, healthy aging, hemodynamics, training

## Abstract

**Background:** Body relaxation and pain reduction are some of the reported benefits of flexibility training (through active stretching exercises), however their effects on posture and blood circulation are uncertain. We aimed to investigate the effects of flexibility training (through active stretching exercises) in combination with multicomponent training (MT) on blood pressure (BP), and the correlation with changes in body alignment and flexibility in physically inactive women.

**Methods:** Women aged 60–70 years were into three groups: multicomponent training group (MT), multicomponent training plus flexibility training group (FT), and control group (CG). After randomization, the resting blood pressure was measured and the participants were reallocated into subgroups according to pressure values >130/80 mmHg (This classification is according to the American Heart Association (AHA), resulting in the subgroups: flexibility training (FT); flexibility training for hypertensive patients (FTSAH); multicomponent training (MT); multicomponent training for hypertensive patients (MTSAH); control group (CG); control group of hypertensive patients (CGSAH). The interventions lasted 14 weeks. Systolic (sBP) and diastolic (dBP) BP, range of motion (flexion and extension), and postural analysis by asymmetry in the frontal plane and asymmetry in the sagittal plane, displacement and the flexibility test were collected before (Pre) and after training (Post). In total, 141 women participated in the study (without SAH: FT = 23, MT = 20, and CG = 21; with SAH: FTSAH = 28, MTSAH = 23, and CGSAH = 26).

**Results:** Systolic blood pressure, in the pre and post moments were: FT (116 ± 6.7 vs. 114 ± 4.7); FTSAH (144 ± 16.5 vs. 121 ± 10.1); MT: (120 ± 6.8 vs. 121 ± 7.3); MTSAH: (137 ± 10.6 vs. 126 ± 13.0); CG: (122 ± 5.3 vs. 133 ± 19.2); and CGSAH: (140 ± 9.7 vs. 143 ± 26.2), presenting an F value (*p*-value - group x time) of 12.00 (<0.001), with improvement in the groups who trained. The diastolic blood pressure in the pre and post moments were: FT (71 ± 4.7 vs. 74 ± 6.8); FTSAH (88 ± 9.6 vs. 70 ± 12.0); MT: (74 ± 4.5 vs. 77 ± 11.7); MTSAH: (76 ± 10.4 vs. 76 ± 10.2); CG: (69 ± 7.11 vs. 82 ± 11.4); and CGSAH: (76 ± 13.4 vs. 86.6 ± 7.7), presenting an F value (*p*-value - group x time) of 8.00 (*p* < 0.001), with improvement in the groups who trained. In the Elastic Net Regression, sBP was influenced by height (β: −0.044); hip flexion (β: 0.071); Shoulder extension (β: 0.104); low back flexion (β: 0.119) and dBP (β: 0.115). In the Elastic Net Regression, dBP was influenced by asymmetry in the sagittal plane variables (0.040); asymmetry in the frontal plane (β: 0.007); knee flexion (β: −0.398); BM (β: 0.007); Shoulder flexion (β: −0.142); Hip flexion (β: −0.004); sBP (β: 0.155) and Ankle Flexion (β: −0.001).

**Conclusion:** The displacement of the asymmetry in the frontal plane and asymmetry in the sagittal plane, and the increase in the flexion position in the hip, lumbar, head, and knee regions, influenced the highest-pressure levels. Multicomponent training associated with flexibility training promoted improvement in body alignment, COM, and joint angles, and decreased blood pressure.

## 1 Introduction

The World Health Organization (WHO) estimates that approximately 600 million people have systemic arterial hypertension (SAH), with a global growth of 60% in cases by 2025, in addition to approximately 7.1 million annual deaths. SAH leads to an increase in the costs to health systems, with an important socioeconomic impact. In Brazil, the prevalence of SAH in older adults aged between 60 and 64 years is 44.4% and among those aged between 65 and 74 years, the value is 52.7% (Malta et al., 2018). According to data from the Ministry of Health (Malta et al., 2018), the prevalence of arterial hypertension was 55.3% in women aged 60–74 years and 60.7% in those aged 75 years and over. One of the main reasons that lead women to have more cardiovascular problems than men is the reduction in endothelial relaxing factors to the detriment of hormonal alterations caused by the non-reproductive phase of women (Boonpim et al., 2017). SAH causes a reduction in quality and life expectancy, especially in the older population (Oliveira Lemes et al., 2017).

During menopause, physiological alterations occur, being marked by a decrease in hormone production and the termination of spontaneous menstruation, which generates transformations in the female organism, predisposing women to an increase in cardiovascular diseases, such as SAH. Changes in the elasticity of body structures, such as blood vessels, joints, and ligaments, lead to loss of functional capacity in women (Kato et al., 2020). Recently, Sobrinho et al. (Sobrinho et al., 2021a) documented that low flexibility is associated with higher levels of BP in older individuals, thus demonstrating a relationship between flexibility and BP values.

SAH is a multifactorial disease and is considered a modifiable risk factor (Nørgaard et al., 2018). The most effective treatment is medication (Funding McManus et al., 2018). However, non-drug interventions (e.g., diet, physical exercise) can significantly reduce the impact of SAH on the health and quality of life of older adults (Logan et al., 2020). Among non-drug strategies, regular physical exercise has been shown to be an important intervention in the control of risks for cardiovascular diseases (García-Hermoso et al., 2020). The increase in physical activity and cardiorespiratory capacity appears to have an effect similar to pharmacotherapy in terms of reducing systolic (sBP) and diastolic (dBP) blood pressure (BP) in patients with arterial hypertension. Reductions of approximately 7 mmHg for sBP and 5 mmHg for dBP have been observed, which can reduce mortality from ischemic heart disease by 7% and from stroke by 10%, in addition to decreasing awake BP and BP in situations of physical and mental stress (Medina et al., 2010).

Although the hypotensive effects that aerobic training provides on BP are already more consolidated, the effects of isolated strength resistance training on BP and arterial stiffness deserve additional clarification (Thomas et al., 2021). The literature shows that when aerobic and strength are combined there is a reduction in BP and optimization of vascular and endothelial muscles (Lima et al., 2017).

In this context, multifunctional training (e.g., combined exercises of localized muscular resistance, strength, and aerobics) can minimize the physiological implications of a sedentary lifestyle, reducing the development and progression of chronic diseases and disabling conditions (Cadore et al., 2014; Pepera et al., 2021).

However, to the best of our knowledge, no studies have examined the hemodynamic effects of multifunctional training associated with flexibility training in older women with SAH. In flexibility training, responses to mechanical stretch and shear stimuli improve muscles and tendons (mechanical shear stress of muscles and tendons), and also have effects on target muscle vasculature, which is important for maintaining vascular function (Hotta et al., 2018; Pepera et al., 2021).

Although the beneficial effects of exercise are known, the rate of adherence to physical training programs is low in people with special conditions (Hotta et al., 2018). Older people and people with obesity may have physical or musculoskeletal limitations, which can reduce their participation in more active or strenuous modalities or in conventional places of physical exercise. The intensity of muscle stretching exercise is relatively light compared to aerobic exercise, so that even the oldest old and/or obese individuals can perform muscle stretching exercise with minimal risk of injury (Lima et al., 2017). Improvements in health parameters, such as hemodynamics, are seen in the literature, separately for flexibility training (through active stretching exercises) and other types of training (Lima et al., 2017; Hotta et al., 2018; Bisconti et al., 2020). Flexibility training through stretching exercises is feasible for these individuals due to its greater safety and clinically relevant reported results (Bisconti et al., 2020). Studies that associate training through stretching exercise with other types of training, such as multicomponent training in hypertensive older women, are important to enhance health outcomes (Lima et al., 2017).

According to a review conducted by Kruse and Scheuermann (2017), skeletal muscle stretching, as a form of low-intensity exercise, has recently been found to induce significant cardiovascular responses in humans. While certain vascular effects, such as arterial stiffness, have been elucidated, the review highlighted numerous other acute and long-term vascular adaptations associated with stretching that still lack conclusive evidence, including the effects on blood pressure.

Knowing that vascular function and arterial stiffness are important markers of cardiovascular health and cardiovascular comorbidity, (Bisconti et al., 2020), considering how to improve and combine these training modalities effectively, or how to enhance these results, is necessary to improve the health of older women.

Regarding postural improvement, to our knowledge, the associations between posture angles and blood pressure data are still limited in the literature. To date, the hypothesis that postural alterations can influence the genesis and maintenance of SAH has been little explored (Bisconti et al., 2020). We hypothesize hypothesis that flexibility training” (through active stretching exercises) can contribute to body flexibility, in addition to postural adjustments, which may influence the vascular system.

## 2 Methods

### 2.1 Study design

**FIGURE 1:**
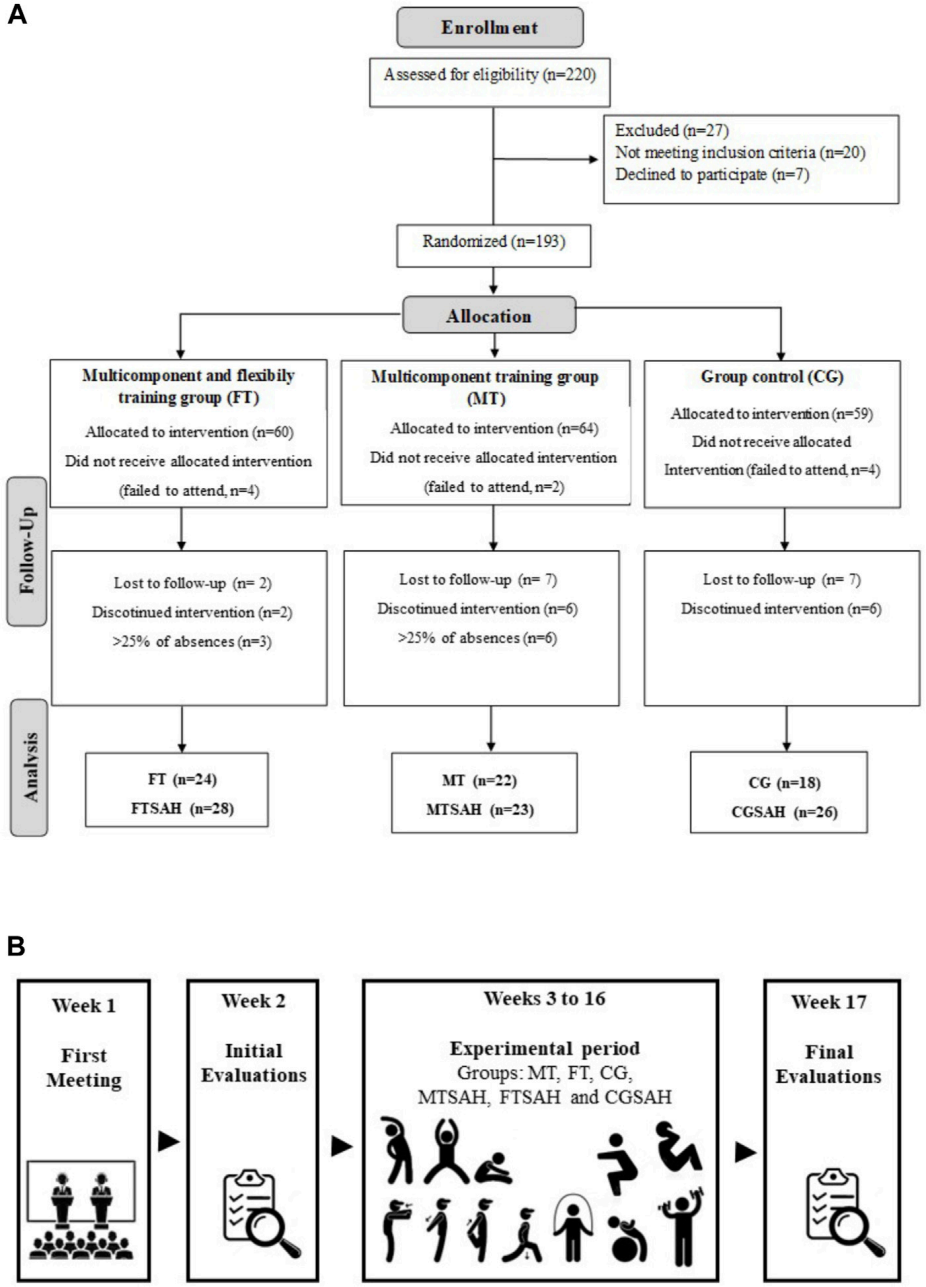
Flowchart and experimental design. Note: FT, flexibility training; FTSAH, flexibility training for hypertensive patients; MT, multicomponent training; MTSAH, multicomponent training for hypertensive patients; CG, control group; CGSAH, control group of hypertensive patients. Figure 1A: flowchart; Figure 1B: sample design.

The experimental procedures were submitted and approved by the Ethics Committee in Research with Human Beings of the School of Physical Education and Sport of Ribeirão Preto, University of São Paulo (CAAE: 63681517.3.0000.5659, 24 February, 2017). Participants who agreed to participate in the study signed a consent form attesting to their participation in the research. Participants were recruited through publicity in local media and social networks. Before the first assessment, the participants were invited to a presentation meeting (Moher et al., 2010), where they received information on the research objective and details about the test protocol - they also signed the informed written consent at the end of the session. After this step, participants were randomized, by a blinded researcher who did not participate in the study evaluations, into three groups: multicomponent training group (MT), multicomponent training plus flexibility training group (FT), and control group (CG). After randomization, the resting blood pressure was measured and the participants were reallocated into subgroups considering 130/80 mmHg as the minimum value to be included in the SAH group (According to the American Heart Association (AHA)), resulting in the subgroups: flexibility training (FT); flexibility training for hypertensive patients (FTSAH); multicomponent training (MT); multicomponent training for hypertensive patients (MTSAH); control group (CG); control group of hypertensive patients (CGSAH) (Figure 1A). Pre- and post-intervention assessments were performed. The experimental design demonstrates the steps in the study, including the recruitment week, evaluation weeks, intervention with a total duration of 14 weeks, and post-intervention evaluation weeks (Figure 1B).

### 2.2 Participants

To be included in the study, the participants should be postmenopausal women, aged between 60 and 70 years, with a medical certificate of clearance to practice physical activity, and physically inactive, according to a score <9.11 in the Modified Baecke Questionnaire for Elderly (MBQE) (Mazo et al., 2001). The exclusion criteria were diseases and/or functional limitations (motor, auditory, or visual deficits) that prevented the performance of the tests and the proposed physical training. Those who missed more than 25% of the training sessions were also excluded from the final sample. We chose to exclude them instead of applying the intention-to-treat analysis to the detriment of a parallel clinical trial design.

During the development of the study, participants underwent a critical cognitive assessment. The Montreal Cognitive Assessment (MoCA) cognitive screening questionnaire was applied to understand the impact of these types of training on the participants’ mental health. Generally, the cut-off value is usually set around 26 to 30 points on a total scale of 30. Values below 26 points may indicate the presence of mild cognitive decline (MCD) or other cognitive changes that warrant attention and further investigation. Thus, Table 3 shows the pre and post-data of the mental analysis variable.

### 2.3 Intervention

#### 2.3.1 Multicomponent training

The training was applied twice a week on non-consecutive days, in order to develop coordinative motor skills and conditioning motor skills. The training lasted 90 min per session, divided into an initial 15 min of warm-up, balance, motor coordination, and games, 35 min of muscle strength training, 35 min of aerobic activities (e.g., running, stationary running, jumping jacks, dancing, volleyball, bocce, adapted basketball), and a final 5 minutes of relaxation (Sobrinho et al., 2021a; Sobrinho et al., 2021b). Training intensity was monitored using the Borg scale, with the objective of tracking the perceived exertion in values from 3 to 10, progressively, on a scale from 0 to 10 (weeks 1 to 2 = 3 to 4, from moderate to mild; weeks 3–5 = 4–6, mild to severe; weeks 6–8 = 6 to 7, severe to very severe; weeks 9–11 = 7 to 8, very severe; weeks 11–14 = 8–10, very intense to the maximum), representing a moderate to high intensity of physical exercise (Borg and Noble, 1974). This training focused on strengthening the following muscles: rectus abdominis, external abdominal oblique, internal abdominal oblique, transversus abdominis, glutes, abductors, knee flexors and extensors, deep neck flexors, serratus, rhomboids, intermediate and ascending trapezius, rotator cuff, and paravertebral or erector spinae.

#### 2.3.2 Individualized flexibility training

Flexibility capacity was trained using the active stretching method with accessories, following the protocol proposed by Nelson and Kokkonen (2007), which adheres to the recommendations of the American College of Sports Medicine (2019), in relation to volume and intensity. Participants were separated into groups for stretching exercise aimed at postural changes (hip flexor muscles, spine extensors, elevators, and scapular protractors), focusing on the individual needs identified after performing the postural analysis. The flexibility training sessions had an average duration that increased gradually over the 12 weeks, starting from 10 min and reaching 40 min per session, as demonstrated in Table 1. The exercise protocol contained three levels of exercise complexity for each postural compensation, with a new complexity being added every 4 weeks. The intensity and volume protocol was divided into four levels, with progression of stretching exercise time and pain perception by the pain scale (Thong et al., 2018). The training was performed twice a week following the training progression in Table 1.

**TABLE 1:** Flexibility training protocol adopted in the intervention.

	Level 1	Level 2	Level 3	Level 4
Intervention week	1-2	3–6	7–10	11–14
Duration of session	10′	20′	30′	40′
Time under tension	10”	15″	20″	25″
Interval between series	10″	15″	20″	25″
Series per exercise	1	2	3	4
Pain level^*^	1 to 3	2 to 4	4 to 6	6 to 8
Exercises per body region^$^	2	3	3	4
Weekly dose^#^	1,200″	2,400″	3,600″	4,800″

Note: *, numeric visual/verbal scale of pain from 0 to 10; $, 8 body regions were worked in each individual - initial evaluations of each participant were considered to choose these regions; #, weekly dose (seconds) = duration of session (min) * 2 (sessions/week) * 60 (seconds/minute).

### 2.4 Assessments

To characterize the participants, a questionnaire developed by the researchers was used to analyze demographic and health data. Heart rate was also measured using an armband with an Omron-HBP-1300 device, only one HR measurement was performed. Measurements of sBP and dBP (mmHg) were performed with an automatic digital arm pressure gauge (OMRON^®^, model HEM-7113, SBH, 2010). Participants rested in a sitting position, with their backs and arms supported and legs uncrossed, in a quiet room for 5 minutes. Then, an operator performed two standard MAP measurements using an appropriately sized cuff with an Omron-HBP-1300 device, with a two-minute interval between each measurement. Thus, we obtained two values at the pre-and post-intervention time points. For these values, the average of the measurements was calculated. Only participants with four complete measurements were included in the final analysis. Systolic and diastolic BP values were analyzed separately. Thus, if a participant, for example, had a difference >10 mmHg between systolic measurements 1 and 2 but ≤10 mmHg between diastolic 1 and 2, we assigned a third and fourth measurement; none of the participants needed the third and fourth measurement, besides all hypertensive participants had controlled disease.

In the anthropometric evaluation, body mass (kg) was collected with a digital scale (Welmy W200 ALCD) with a 50-g scale and stature (m) with a stadiometer with a precision of 1 millimeter (Balmak - EST-223). Data collection was performed according to Lohman’s recommendations (Lohman and Martorell, 1998).

#### 2.4.1 Motor assessments and assessment tools

Flexibility was analyzed by the hands-on-back and sit-and-reach tests, both taken from the Rikli and Jones test set (Rikli and Jones, 2008). In the hands-on-back test, participants were asked to place their hands behind their backs on both sides twice in a row and then asked to choose their preferred side for measurement. After choosing the side, the distances between the fingertips were measured. Two attempts are usually made to obtain an accurate size, and the farthest value is recorded as the test result.

In the sit-and-reach test, the participants were positioned sitting with one leg extended and the other bent at 90°. They were instructed to reach as far as possible towards the foot of the extended leg, keeping the knees extended. During the execution, they performed this movement on both sides twice in a row, and then the participants were asked to choose the side of their preference for measurement. Similarly, two trials were performed to obtain a more reliable assessment of flexibility, and the furthest result reached by the fingertips was recorded as the test value.

In the study context, the SAPo (Postural Analysis System) biophotogrammetry software was used to conduct the analyses. SAPo is a specialized software designed to assess posture and body movement in different planes, allowing for a comprehensive and detailed analysis (Barbosa, 2001).

Analysis of asymmetry in the frontal plane by SAPo involves using information from images or videos to examine the body’s alignment about the frontal plane. This plane is an imaginary division separating the body into left and right halves. The software can identify discrepancies in weight distribution and the positioning of the upper and lower limbs on each side of the body (Barbosa, 2001).

The analysis of asymmetry in the sagittal plane by SAPo focuses on assessing the body’s center of gravity during movement. The sagittal plane is also an imaginary division separating the body from the left and right sides. Still, it is perpendicular to the frontal plane, passing through the body’s midline. The software can calculate and compare the position of the center of gravity between the left and right sides, detecting asymmetries that may exist (Barbosa, 2001).

The analysis of asymmetry in the frontal plane and asymmetry in the sagittal plane were measured by biophotogrammetry. The asymmetry in the frontal plane and asymmetry in the sagittal plane (Center Gravity) is a variable that can be measured using computerized photogrammetry, and its trajectory is a measure used to understand the mechanisms of postural control in different motor actions. The asymmetry in the frontal plane and asymmetry in the sagittal plane is defined as the point of application of the resultant gravitational force on the body acting on the base of support, which is delimited by the lateral edges of the feet. Biofotogrammetry programs typically estimate the center of mass by analyzing the distribution of mass within the body segments.

We used a digital photographic camera positioned on a level and upright tripod with a height of 95 cm from the ground and at a distance of 3 m from the participant, as this is suggested as the best position for recording in the literature (Barbosa, 2001; Porto and Okazaki, 2017). A plumb line with two Styrofoam balls, 1 m apart, was placed next to the participant. This distance was used as a calibrator, according to the protocol of the SAPO^®^ program. The participants took pictures with bikinis to better visualize the anatomical structures, where anatomical markers were placed at 32 body points. The images were taken in frontal, coast, right, and left side views. The protocol has a total of 32 points, which can be analyzed from different photographic views (Barbosa, 2001).

To measure the joint range of motion of the older women, a goniometer was used, which analyzes joint flexibility in the cervical, shoulder, lumbar, hip, knee, and ankle regions, in flexion and extension movements. These movements are more altered by the declines of aging (Norkin and White, 1995).

### 2.5 Statistical analysis

The data obtained were organized in a double-entry database, using the Excel^®^ version 2013 program. Data are presented as mean and standard deviation. To assess the normality of the data, the Anderson-Darling test was used, and the variances were analyzed using the Levene test. We performed repeated measures one-way ANOVA (ANOVA1) and two-way ANOVA (ANOVA2), with Tukey’s post-hoc, for the participants’ characterization and descriptions of physical activity, anthropometric data, and blood pressure. The size of the Cohen d effect was calculated, considering the size of the effect as medium (0.50–0.79), large (0.80–1.29), and very large (>1.30).

Multiple linear regression was performed using the fractional change (delta) of both independent variables and predictors. The delta variation (Δ) (Δ = Post-training - Pre-training/Pre-training) between pre- and post-intervention moments was used to quantify the variation in quantitative variables. We used multiple linear regression to analyze which of the variables studied may have an influence on blood pressure.

The predictor variables used in the initial entry of the elastic net as a baseline were age, height, and years of schooling. Body mass, BMI, sit and reach, hands on the back, systolic and diastolic blood pressure, range of motion, and postural variables were used as fractional changes in the elastic net regression input. The dimensionless fractional change variables of systolic and diastolic blood pressure were used for the elastic net regression statistical analysis output variables.

We included the fractional change values of diastolic blood pressure (dBP) as a predictor of systolic blood pressure (sBP) and conversely, in the elastic net regression statistical analyses. Thus, we recognize the existence of a relationship between these variables. Blood pressure consists of two values: systolic blood pressure, which is the highest value recorded during heart contraction, and diastolic blood pressure, which is the lowest value recorded when the heart is relaxed between beats. Both values are crucial in assessing cardiovascular health and are significantly related. Therefore, analysis of the behavior of this variable, which is already expected to be influential, provides an additional parameter of data reliability.

“Data were analyzed using the ststistical program Statistical Package for the Social Sciences (SPSS) version 20.0, Microsoft Excel, and StatFi (AnalystSoft).

Elastic Net was used to select independent predictors and estimate their effects on systolic and diastolic blood pressure. Elastic Net is a machine learning algorithm for smoothing regression models combining Ridge and Lasso, which balances model selection so that the model is neither too complex (i.e., overfitted) nor too simple (i.e., under fitted) (Friedman et al., 2010). In other words, Elastic Net fits a model with all supplied variables (sometimes called characteristics) but constrains the coefficients by reducing those that are not informative to zero. The selection of lambda (λ) was based on Bayesian Information Criterion (BIC). Different lambda values were tested, and the Elastic Net model was fitted for each value. Then, the BIC was calculated for each fitted model. The lambda value that minimized the BIC was considered optimal for the Elastic Net model as it balanced model fit and complexity. The Excel software package provides built-in functions that automatically determine the optimal lambda based on this method. This automated procedure helps researchers select an appropriate lambda value without manual trial and error.

The sBP and DBP data were split into two datasets using random sampling with a ratio of 2:1. For each α value, we applied the corresponding model to our test dataset to predict the outcome. When α = 0, elastic net does a pure ridge regression. When α = 1, elastic net does a pure lasso regression.

We then calculated each model’s mean square error (MSE) or misclassification rate. We stated that the best model was the one with the lowest MSE. In the best model, the coefficient estimates for certain variables indicate that those variables are robust predictors of the outcome. Variables without a coefficient estimate are not considered robust predictors of the outcome. Our primary analyses were to determine which variables best predicted i) the influence on systolic blood pressure outcomes and ii) the influence on diastolic blood pressure outcomes. The predictors we included in the Elastic Net models were age, years of schooling, height, body mass, BMI, sit-and-reach, hands-on-back, and predictor postural variables. Due to different rates of missing data, sample sizes varied when including different variables in the model. We standardized the scales of all predictors to provide standardized Elastic Net model coefficients.

The statistical analysis was performed using a standard software package, and the results are presented in accordance with established statistical guidelines (Friedman et al., 2010). The statistical significance for the differences between the pre and post-groups was set at *p* < 0.05, indicating a significance level (alpha) of 5%.

## 3 Results

In the context of statistical analysis, an initial focal point pertains to Supplementary Table S1, wherein the behavior of the data is meticulously examined. Within the framework of the Anderson analysis, it is noteworthy that the Darling test outcomes indicate a departure from normal distribution in the blood pressure values.

In the analysis, 64 women without systemic arterial hypertension were included, 23 in the FT, 20 in the MT, and 21 in the CG groups, and 77 women with SAH, 28 women in the FTSAH, 23 in the MTSAH, and 26 in the CGSAH. There were no statistical differences between the groups in the mean age (65.4 ± 5.2 years, considering all groups), years of schooling (10.8 ± 4.4 years, considering all groups), and age of the last menstrual cycle (48.1 ± 5.8 years, considering all groups) (Table 2).

**TABLE 2:** Characterization of individuals by mean and standard deviation.

Variable	FT (n = 23)	FTSAH (*n* = 28)	MT (*n* = 20)	MTSAH (*n* = 23)	CG (*n* = 21)	CGSAH (*n* = 26)
Age (years)	64.4 ± 2.3	65.1 ± 4.0	65.3 ± 5.0	65.3 ± 3.9	66.1 ± 4.9	66.8 ± 5.4
Years of schooling (years)	11.3 ± 5.44	10.8 ± 5.7	10.2 ± 3.4	11.2 ± 3.5	9.7 ± 3.0	11.1 ± 3.2
Last menstrual cycle (age)	47.9 ± 6.2	48.3 ± 6.2	48.2 ± 4.3	47.2 ± 4.5	47.8 ± 5.0	48.1 ± 7.2

Note–FT, flexibility training; FTSAH, flexibility training for hypertensive patients; MT, multicomponent training; MTSAH, multicomponent training for hypertensive patients; CG, control group; CGSAH, hypertensive control group; *p* < 0.05 for differences between pre and post groups (ANOVA, one-way test was used for repeated measures, followed by Tukey Post-Hoc).

There was group and time interaction in the MBQE variable, showing improvements in the FT and FTSAH groups (Table 3).

**TABLE 3:** Characterization of anthropometric data and level of physical activity before and after intervention by mean and standard deviation.

Variable	Group (n)	Pre	Post	F/*p*-value (group)	F/*p*-value (time)	F/*p*-value (group x time)
BM (kg)	FT (24)	70.7 ± 9.3	68.0 ± 13.8	2.050/0.076	0.011/0.915	2.309/0.047
	FTSAH (28)	76.2 ± 15.3	76.0 ± 13.6			
	MT (22)	72.6 ± 12.8	71.0 ± 11.1			
	MTSAH (23)	77.4 ± 15.0	76.11 ± 15.0			
	CG (18)	73.9 ± 16.13	81.3 ± 14.9			
	CGSAH (26)	77.8 ± 14.4	77.9 ± 13.5			
STATURE (cm)	FT (24)	1.57 ± 0.0	1.58 ± 0.0	0.614/0.690	0.003/0.955	3.152/0.010
	FTSAH (28)	1.59 ± 0.6	1.58 ± 0.0			
	MT (22)	1.60 ± 0.0	1.59 ± 0.6			
	MTSAH (23)	1.54 ± 0.0	1.55 ± 0.0			
	CG (18)	1.58 ± 0.0	1.57 ± 0.0			
	CGSAH (26)	1.59 ± 0.0	1.58 ± 0.0			
MBQE (point)	FT (24)	6.5 ± 10.1	12.9 ± 5.2^*^	1.0/0.381	61.0/<0.001	6.9/<0.001
	FTSAH (28)	6.9 ± 6.3	14.2 ± 5.8^*^			
	MT (22)	6.5 ± 9.2	8.9 ± 7.2^*,a,b^			
	MTSAH (23)	6.7 ± 8.9	11.1 ± 8.4			
	CG (18)	6.4 ± 8.1	7.9 ± 7.2			
	CGSAH (26)	7.0 ± 11.7	8.3 ± 11.8			
HEART RATE (bpm)	FT (24)	72.9 ± 3.6	72.9 ± 3.6	1.220/0.303	0.093/0.760	0.519/0.761
	FTSAH (28)	72.1 ± 3.9	72.1 ± 3.6			
	MT (22)	72.8 ± 4.2	73.3 ± 3.9			
	MTSAH (23)	72.3 ± 3.8	71.8 ± 3.4			
	CG (18)	72.8 ± 3.9	72.9 ± 3.9			
	CGSAH (26)	71.1 ± 3.9	70.9 ± 3.3			
MoCA (point)	FT (24)	18.5 ± 2.6	23.8 ± 1.3	0.003/0.493	78.0/<0.001	4.7/<0.001
	FTSAH (28)	18.9 ± 2.4	25.2 ± 2.5			
	MT (22)	19.4 ± 4.7	21.4 ± 3.7			
	MTSAH (23)	19.01 ± 2.7	22.7 ± 4.6			
	CG (18)	19.1 ± 3.1	17.8 ± 5.4			
	CGSAH (26)	20.4 ± 3.9	20.9 ± 2.1			

Note–FT: flexibility training; FTSAH: flexibility training for hypertensive patients; MT: multicomponent training; MTSAH: multicomponent training for hypertensive patients; CG: group control; CGSAH: hypertensive control group; BM: body mass; MBQE: modified baecke questionnaire for elderly; MoCA: Montreal Cognitive Assessment *p < 0.05 for differences between pre and post groups (two-way ANOVA, test was used for repeated measures, followed by Tukey Post-Hoc; a: FT, difference at the same time; b: FTSAH, difference at the same time.

**FIGURE 2:**
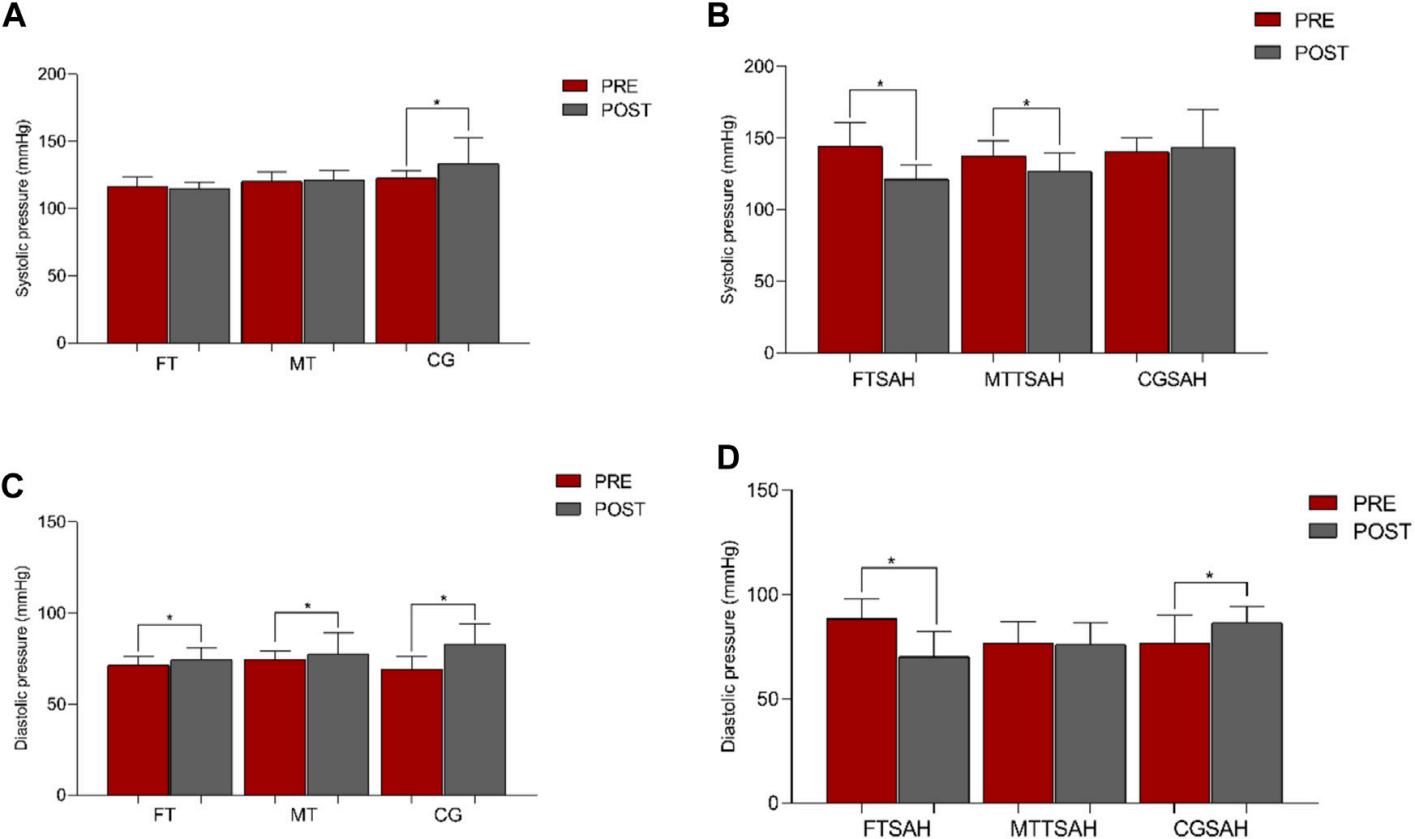
Systolic and diastolic blood pressure values in the pre and post moments of each analyzed group. Note: FT, flexibility training; FTSAH, flexibility training for hypertensive patients; MT, Multicomponent Training; MTSAH, multicomponent training for hypertensive patients; CG, control group; CGSAH, hypertensive control group; sBP, Systolic blood pressure; dBP, Diastolic blood pressure; *p < 0.05 for differences between pre and post groups (two-way ANOVA test was used for repeated measures, followed by Tukey Post-Hoc).

The group with SAH that trained flexibility associated with multicomponent training showed improvement after the intervention in both systolic and diastolic pressure. The MTSAH group presented improvement only in systolic blood pressure (Figure 2).

The comparison of sBP values with the repeated measures test showed values of F = 12.0 and *p* < 0.001 (group x time). The mean and standard deviation values of sBP in the pre and post groups were: FT (116 ± 6.7 vs. 114 ± 4.7, Cohen’s d = 0.34); FTSAH (144 ± 16.5 vs. 121 ± 10.1, Cohen’s d = 1.68); MT: (120 ± 6.8 vs. 121 ± 7.3, Cohen’s d = −0.14); MTSAH: (137 ± 10.6 vs. 126 ± 13.0, Cohen’s d = 0.92); CG: (122 ± 5.3 vs. 133 ± 19.2, Cohen’s d = −0.78); and CGSAH: (140 ± 9.7 vs. 143 ± 26.2 Cohen’s d = −0.15) (Table 4).

**TABLE 4:** Table with mean and standard deviation of systolic and diastolic blood pressure at pre and post-intervention, with d'Cohen’s effect size analysis.

Group	sBPpre (mmHg)	sBPpost (mmHg)	Cohen’s d	Effect
FT (24)	117 ± 6.7	114 ± 4.7	0.34	Small
FTSAH (28)	145 ± 14.9	121 ± 10.1	1.68	Very large
MT (22)	120 ± 6.9	121 ± 7.3	−0.14	Insignificant
MTSAH (23)	136 ± 10.0	126 ± 13.0	0.92	Large
CG (18)	122 ± 4.5	133 ± 19.2	−0.78	Medium
CGSAH (26)	142 ± 9.0	143 ± 26.2	−0.15	Insignificant

Group	dBPpre (mmHg)	dBPpost (mmHg)	Cohen’s d	Effect
FT (24)	72 ± 4.7	74 ± 6.8	−0.51	Medium
FTSAH (28)	89 ± 8.0	70 ± 12.0	1.65	Very large
MT (22)	74 ± 4.6	77 ± 11.7	−0.33	Small
MTSAH (23)	76 ± 9.1	76 ± 10.2	0.00	Insignificant
CG (18)	70 ± 6.1	82 ± 11.4	0.31	Small
CGSAH (26)	78 ± 11.3	86.6 ± 7.7	−0.18	Insignificant

Note–FT: flexibility training; FTSAH: flexibility training for hypertensive patients; MT: multicomponent training; MTSAH: multicomponent training for hypertensive patients; CG: control group; CGSAH: hypertensive control group; size of the effect as medium (0.50–0.79), large (0.80–1.29), and very large (>1.30).

The analysis of dBP values with the repeated measures test showed values of F = 8.0 and *p* < 0.001 (group × time effect). The mean and standard deviation values for DBP of the groups between pre and post intervention study were: FT (71 ± 4.7 vs. 74 ± 6.8, Cohen’s d = −0.51); FTSAH (88 ± 9.6 vs. 70 ± 12.0, Cohen’s d = 1.65); MT: (74 ± 4.5 vs. 77 ± 11.7, Cohen’s d = −0.33); MTSAH: (76 ± 10.4 vs. 76 ± 10.2, Cohen’s d = 0.00); CG: (69 ± 7.11 vs. 82 ± 11.4, Cohen’s d = 0.31); and CGSAH: (76 ± 13.4 vs. 86.6 ± 7.7, Cohen’s d = −0.18) (Table 4).

The mean and standard deviation values of the flexibility, goniometry, and asymmetry in the frontal plane and asymmetry in the sagittal plane variables are available in Tables 1–3 of the Supplementary Material.

We used Elastic Net, a machine learning approach, to select the most robust factors for predicted i) the influence on systolic blood pressure outcomes and ii) the influence on diastolic blood pressure outcomes. We tested the following variables as potential predictors: age, years of schooling, height, body mass, BMI, sit-and-reach, hands-on-back, and predictor postural variables.

In particular, we found the following predictors to be important for the influence of systolic blood pressure, in order of greatest to least magnitude of effect: Low back flexion, dBP, Shoulder extension, Hip flexion and Height (Table 5). Regarding the influence of diastolic blood pressure, we found the following robust predictors, in order from largest to smallest effect size: asymmetry in the frontal, Ankle flexion, Hip flexion, BM, Shoulder flexion, Knee extension, and Knee flexion (Table 5). Effect magnitude was performed based on the standardized Elastic Net coefficients (Table 5).

**TABLE 5:** Robust systolic and diastolic blood pressure predictors from Elastic Net (*N* = 144, α = 0.3). The variables with corresponding coefficients are considered essential predictors of perceived systolic and diastolic blood pressure. The predictors that indicated a coefficient approaching 0 are non-significant predictors to the final model and were not entered into the table.

Systolic blood pressure (sBP)		Diastolic blood pressure (dBP)	
Variable	Standardized coefficient	Variable	Standardized coefficient
Height	−0.044	BM	0.007
Shoulder extension	0.104	Shoulder flexion	−0.142
Low back flexion	0.119	Hip flexion	−0.004
Hip flexion	0.071	sBP	0.155
dBP	0.115	Knee flexion	−0.398
		Ankle Flexion	−0.001
		Frontal Plane Asymmetry	0.007
		Asymmetry in the Sagittal Plane	0.040

Note: sBP, lambda: 0.023 and sBP, Alpha: 0.980; dBP, lambda: 0.132 e dBP, alpha: 0.162; BM, body mass; BMI, body mass index.

After performing the Elastic Net analysis, we obtained the estimated coefficients for the selected independent variables. The results revealed a combination of variables relevant to the systolic and diastolic blood pressure predictive model. In the Elastic Net model, two regularization parameters were used, sBP lambda: 0.023 and sBP alpha: 0.980; dBP lambda: 0.132 and dBP alpha: 0.162. The parameter α = alpha controls the balance between the Lasso and Ridge penalty, while the parameter λ= lambda controls the level of penalty applied to the coefficients.

The estimated coefficients for the independent variables indicated the direction and magnitude of each variable’s influence on the dependent variable. For example, for the sBP variable, the estimated coefficient was 0.119, indicating a positive relationship with the Low back flexion variable. For the variable height, the estimated coefficient was −0.044, suggesting a negative relationship.

For sbp, Table 5 indicates that the standardized coefficient for height is −0.044. This means a 1-SD increase in height is associated with a −4.4% change in sBP, from pre to post. The standardized coefficient for low back flexion is +0.119. This means a 1-SD increase in the fractional change in the low back flexion angle is associated with a +11.9% increase in sBP, from pre to post.

As for dBP, Table 5 indicates that negative standardized coefficients for joint mobilities suggest that an improvement in the range of motion tends to reduce diastolic blood pressure. Specifically, for knee flexion, the coefficient is −0.398. This means a 1-SD increase in knee flexion is associated with a −39.8% change in dBP, from pre to post.

Furthermore, it is essential to note that the regularization applied by Elastic Net reduced the number of selected variables. Through the combination of Lasso and Ridge penalties, the model could automatically select relevant variables, eliminating those with less impact on the dependent variable. This contributed to the simplification and interpretability of the model.

## 4 Discussion

In our results, we observed that the displacement of asymmetry in the frontal plane and asymmetry in the sagittal plane, body mass and modification of flexion angles in the hip, shoulder, ankle and knee regions were associated with modulations in the deltas of dBP pressure values as an effect of physical training. Height and changes in shoulder extension, lumbar flexion and hip flexion angles were associated with modulation of the deltas of sBP blood pressure values. Participants with SAH who exercised in the FT group associated with TM showed greater reductions in sBP and dBP values. On the other hand, participants with SAH who performed only the MT showed reductions only in sBP, showing that flexibility training is important to improve the adjustment in the postural pattern of women with SAH.

In the present study, heart rate was not measured during individual training sessions; it was assessed during pre- and post-intervention assessments. In this study, no significant changes in heart rate were observed through the statistical analysis of the results. Despite the lack of statistical significance, including these data contributes to a comprehensive understanding of the participant’s physiological responses to the intervention. It should be noted that these results diverge from the existing literature.

For example, Mueck-Weymann et al. (2004) reported increased muscle flexibility and a significant positive impact on heart rate variability (HRV) among healthy male athletes who followed a standardized 15-min stretching program over 28 days. In addition, Papp et al. (2013) demonstrated a significant improvement in HRV after 8 weeks of hatha yoga practice among older adults, indicating an increase in vagal tone and a reduction in sympathetic activity.

To the best of our knowledge, associations between postural angles and blood pressure data are still limited in the literature, and the hypothesis that postural changes and changes in movement amplitudes may influence the genesis and modulation of SAH is still poorly explored to date (Goes et al., 2018; Cadore et al., 2019).

Excessive adipose tissue and physical inactivity are independent risk factors for the deterioration of vascular function and contribute to the onset of cardiovascular disease and reducing flexibility (Cadore et al., 2014; Cadore et al., 2019). Central obesity and anterior trunk and hip flexion displacement favor forward trunk projection. As a possible attempt to compensate for posterior body displacement, dominant laterality deregulates the body laterally. All these compensations are observed with the aging process (Goes et al., 2018). These changes were associated with a more significant variation in blood pressure in our study. The anterior displacement of the trunk may have some reflection on the vestibular system, as it plays a vital role in adjusting the blood distribution in the body during movement and posture changes (Goes et al., 2018).

The study by Pollak et al. (1976) investigated postural changes and their influence on blood pressure. A difference in blood pressure was observed between individuals with different weights, higher in heavier individuals. In addition, the study highlighted differences in blood pressure in other regions of the body, mainly in the ankle and shoulder, due to gravitational effects and overload on the structures of the extremities close to the ground.

Misaligned body positioning can stimulate the otolithic organs, which can promote muscle sympathetic nerve activity (SNA), with a consequent increase in peripheral vascular resistance (PVR) and cardiac output (CO) (González-Sánchez et al., 2014). In addition, there appears to be an additive interaction between ANS and BP when the musculoskeletal and sympathetic-vestibular reflexes are activated simultaneously (Ray and Carter, 2003). The literature also points out that greater activation of the motor system can lead to greater activation of the noradrenergic system, with a consequent increase in the stimulation of sympathetic tone, which maintains postural adjustments. This hyperstimulation of the sympathetic nervous system (SNS) by posture adds to the hyperstimulation of the SNS by SAH, and postural changes may generate an incremental effect on SAH (Goes et al., 2018).

Hip, lumbar, shoulder, ankle, and knee assessments were associated with dBP and sBP. Changes in muscle tension state can generate mechanical compression on the outer vessel wall, which favors adrenaline release and increased noradrenergic sensitivity, with persistent perfusion reduction, compromising muscle performance (Mota et al., 2008). Sustained muscle contraction can increase tension in the outer vessel wall, transmitting this mechanical shear force to the inner face of the vessel wall (Goes et al., 2018).

The increase in peripheral vascular resistance and cardiac output, in an attempt to restore muscle blood flow, favors elevated blood pressure levels (Chicayban and Malagris, 2014). Studies show that it is possible to understand that altered posture, sustained for a long time, can stimulate the metaboreceptors of large muscle groups, such as tibialis anterior, triceps sural, and hamstrings, causing an increase in BP and a decrease in perfusion pressure. Changes in these regions were also prominent in the modulation of diastolic pressure in the data of this study. Some resulting consequences are early muscle fatigue and decreased muscle performance, which are also associated with the changes existing during aging (Chicayban and Malagris, 2014; Goes et al., 2018).

Our results agree with previous studies that showed that joint mobility of movement and postural change by stretching for 14 weeks effectively reduces blood pressure. Our analyses identified that individuals with SAH benefit the most from this training method to lower blood pressure values. Previous studies have shown that the association of flexibility training with multicomponent training led to improved postural alignment and, thus, improved performance in several physical capacities (Sobrinho et al., 2021a; Sobrinho et al., 2021b). Due to these factors, physical exercise has been considered one of the main strategies to reduce morbidity and mortality in patients with SAH, in addition to reducing public health expenditure (Quesada-Valle et al., 2018). Increasingly, the importance of flexibility training to improve health-related physical abilities is highlighted by the ACSM recommendations.

Recently, Ko et al. (2020) demonstrated that body flexibility can reduce blood pressure. Unlike other studies, which only showed the benefits of stretching exercise, this research made a comparison with walking, which is a physical exercise already known to reduce BP (Quesada-Valle et al., 2018). The authors divided elderly individuals (16 men and 24 women) into two groups: one group followed a routine of 30 min of daily stretching exercises for 5 days, and the other performed a short walk for the same time (Pollak et al., 1976). After 8 weeks, the group that practiced stretching exercises showed a more significant reduction in blood pressure (*p* < 0.05) for sitting systolic BP (146 ± 9 to 140 ± 12 vs. 139 ± 9 to 142 ± 12 mmHg) and supine diastolic BP (85 ± 7 to 78 ± 8 vs. 81 ± 7 to 82 ± 7 mmHg). In contrast, subjects who walked showed more significant reductions in waist body fat. In our study, hypertensive individuals who practiced flexibility also showed better results (Ko et al., 2020).

The results of analysis revealed a combination of variables relevant to the predictive model of systolic and diastolic blood pressure. The estimated coefficients obtained from the study provided valuable information about the direction and magnitude of the influence of each independent variable on the dependent variables.

The systolic blood pressure (sBP) variable, the estimated coefficient of 0.119, indicated a positive relationship with the lumbar flexion variable. This result suggests increased lumbar flexion is associated with higher systolic blood pressure. This result aligns with previous research showing a positive association between spinal flexion and blood pressure. For example, a study by Arima et al. (2019) found that individuals with greater spinal flexion had higher systolic blood pressure readings than individuals with lower flexion angles (Arima et al., 2019).

The study by Pollak et al. (1976) investigated postural changes and their influence on artery pressure, presenting data showing reductions in blood pressure during flexion of the hip and knee joints due to a possible torsion effect of the arteries. These changes in blood pressure in the regions were independent of the weight-height ratio of the subjects. Our data show this behavior in the participants’ weight and the angles of joint flexions.

Regarding diastolic blood pressure (DBP), we observed a prevalence of negative relationships in six variables. Specifically, three variables related to flexion angles and one variable related to extension angles showed negative coefficients. A Fukuda et al. (2020) study found that subjects with higher flexion angles tended to have lower diastolic blood pressure readings (Kuciene and Dulskiene, 2019; Fukuda et al., 2020).

Another study looked at intramuscular pressure at different hip and knee flexion angles. A significant increase in ankle diastolic blood pressure was observed at different joint flexion angles, including the prone position and 45/45 and 90/90° flexion (Leek et al., 2007). These increases in blood pressure may be attributed to local compressive forces due to dependent weight bearing, which redirects blood flow and creates regional hypoperfusion, counteracting the protective effects of elevated blood pressure in these positions. These data suggest a possible influence of joint angulation on blood pressure modulation, corroborating the benefits observed in improved range of motion and highlighting diastolic pressure for this modulation (Leek et al., 2007).

Active postural maneuvers were advocated in the study of Pochet et al. (1993), indicating that they can induce immediate variations in blood pressure, followed by regulation. The study used a wide-range movement to demonstrate “torsion” of the femoral arteries. In the case of the squatting maneuver in the study, initial hypertension can be explained by an increase in cardiac filling pressure, leading to the rise in systolic output by the Frank-Starling mechanism (Pochet et al., 1993). Thus in our results, a part of this response can also be attributed to the “torsion” of the main arteries permeating the hip and lumbar.

The literature, although limited in this regard for discussion, presents evidence that the change in posture significantly impacts diastolic blood pressure. Studies that analyzed pressure changes in different positions, such as lying, standing, and sitting, showed an average increase of 12 mm Hg in the diastolic variable. The systolic pressure showed a change of only 3 mm Hg (Pickering, 1990; Leek et al., 2007). This difference in diastolic blood pressure was also observed in other research, where standing resulted in an average increase of 16 mm Hg, compared to about 9 mm Hg in normotensive subjects and patients with persistent hypertension. This phenomenon is attributed to reduced cardiac output and exaggerated vasoconstriction, maintaining systolic pressure at the expense of increased diastolic pressure (Pickering, 1990). Despite the ease of measurement, the clinical significance of postural changes in blood pressure has received little attention and is observed only in abrupt changes.

In the study conducted by Macwilliam (1933), it was observed that flexion postures such as squatting result in changes in heart rate, showing a possible relationship with the carotid sinus reflex and compression of tissues and vessels. The posture and vascular condition of the lower limbs, bent trunk and knees, crossed legs, and trunk and hip flexion cause a noticeable increase in systolic and diastolic blood pressures. These effects are not mediated by changes in overall blood pressure, suggesting that they depend on afferent impulses related to thigh position and possibly originate from some part of the vascular apparatus under the influence of the hydrostatic factor (Macwilliam, 1933).

Yamamoto et al. (2009) showed that among older people, central aortic stiffness was lower in individuals with high trunk flexibility than in those with low flexibility. Our study also presented a 14-week stretching program, showing improved flexibility, joint amplitude in the trunk and hip region, and blood pressure. Similarly, the survey by Cortez-Cooper et al. (2008) (Kazuhiro et al., 2021) demonstrated that a 12-week stretching program intervention increased joint mobility and flexibility. It decreased carotid pulse pressure and arterial stiffness in middle-aged and elderly adults, corroborating our data showing this behavior in a higher training volume.

On the other hand, the estimated coefficient of −0.044 for the height variable indicated a negative relationship with systolic blood pressure. This suggests that taller individuals tend to have lower systolic blood pressure. This result is consistent with existing literature, which consistently shows an inverse association between Height and blood pressure. A cross-sectional study by Kuciene and Dulskiene (2019) confirmed that taller individuals generally have lower systolic blood pressure levels.

Regarding systolic blood pressure (sBP), we found four variables that showed positive relationships. Of these, two variables were related to flexion angles, one to extension angles and one to diastolic blood pressure. These findings suggest that increased variables are associated with increased systolic blood pressure. These results are consistent with previous research demonstrating positive associations between spinal posture and systolic blood pressure. For example, a study by Bracher et al. (2013) reported that increased flexion angles and higher diastolic blood pressure were associated with elevated systolic blood pressure levels (Bracher et al., 2013).

There is evidence that an increased range of motion can cause changes in physiological systems, such as the reduction of painful symptoms, increased pressure pain threshold, decreased muscle tension and stiffness, increased peripheral blood flow, and reduced blood pressure, among other benefits (Uiterwaal et al., 2003).

The study by Uiterwwal et al. addressed the relationship between blood pressure and tissue stiffness, highlighting that research of this type is mainly limited to the cardiovascular system. This issue raised reinforces the justification for conducting our study. Elvan-Taşpinar et al. (2005) explains in his paper that arterial wall stiffness is determined early in life by mechanisms controlling connective tissue metabolism, which may be true for other tissues in the body, suggesting the hypothesis of this relationship and influence. Therefore, joint mobility, which can be measured non-invasively, may reflect the phenotypic stiffness of various body parts.

In addition to this relevant discussion, the authors presented that healthy people with stiffer joints and skin have higher blood pressure, independent of various confounding factors. The study by Król et al. (2022), which also investigated the range of motion and blood pressure modulation, demonstrated the influence of reduced joint mobility on blood pressure in pregnant women, suggesting a constitutionally determined stiffness of connective tissues.

Arterial wall stiffness is related to blood pressure and is influenced by connective tissue structure. A review from 2022 (Brown and Davis, 2023) demonstrated the effect of stretch on collagen formation and degradation. Connective tissue composition, dominated by type I and III collagens, is similar in ligaments, tendons, capsules, and arterial walls (Brown and Davis, 2023). This may explain the associations between joint mobility and blood pressure (constitutional stiffness).

Limited research suggests that differences in connective tissue may explain the results found. The study by Elvan-Taşpinar et al. (2005), showed that the relationships between blood pressure, tissue flexibility, and joint mobility are not limited to aging patients or those with genetic or metabolic diseases, such as our elderly. In their study of healthy children, the data showed that the greater the flexibility (mobility) in the joints and skin, the children had lower blood pressure and pulse pressure levels, indicating less elastic arterial walls.

A point to be highlighted is that the association between diastolic blood pressure and joint mobility still needs to be fully understood due to the limited literature available for debate on this topic.

The approach of our study is fundamental, as stretching exercises to improve flexibility capacity can cover individuals with more significant mobility complications and is preferred by older adults due to the low discomfort caused by this training method. Thus, it is possible to reduce the arteries’ stiffness, causing less blood flow resistance. Although these results are essential, we strongly recommend adding flexibility training to other physical activities (Ko et al., 2020; Sobrinho et al., 2021a; Sobrinho et al., 2021b).

This work presents some limitations that should be considered. Firstly, it is important to highlight that the participants’ characteristics were limited, as the study focused exclusively on a specific population of physically inactive women aged between 60 and 70 years. Therefore, the generalization of the findings to other age groups, genders, and activity levels may be limited. Additionally, the sample size in each group was relatively small, which can affect the statistical power, overfitting data dredging, and the applicability of the findings to a broader context. It is worth noting that the intervention duration was only 14 weeks, which, although providing valuable insights, may not have fully captured the long-term effects of flexibility and multicomponent training.

Given these limitations, it is recommended that future studies aim to include more diverse populations, encompassing different age groups, genders, and individuals with varying activity levels and health conditions. This would contribute to a greater generalization of the results and provide a more comprehensive understanding of the effects of flexibility and multicomponent training in different population groups. Furthermore, long-term follow-up assessments are essential to determine the persistence and sustainability of the observed improvements in blood pressure and body alignment. Extended follow-up periods would yield valuable information about the long-term benefits and potential risks associated with these interventions, which is a necessity for future studies.

An important limitation of this study is the possibility that the differences observed between the flexibility training (FT) and multicomponent training (MT) groups may not really due to the flexibility training itself but rather the longer total training time in each training session. Both groups had 90 min of multicomponent training per session. The FT group had an additional time of flexibility training since we preferred keep the same MT for both groups.

Additionally, it is suggested that future studies aim to explore the underlying mechanisms by which flexibility and multicomponent training influence blood pressure, body alignment, and joint angles.

Another point to consider is sarcopenia, a noticeable aspect of aging with a higher prevalence in females, directly linked to postural adjustment. Considering future research that analyzes sarcopenia in conjunction with these findings becomes essential (Brown and Davis, 2023). Sarcopenia is a critical aspect to be taken into account when discussing the overall health of older individuals, and its connection with the postural pattern adds valuable context to our study’s blood pressure findings, providing a potential perspective (Smith and Johnson, 2020; Brown and Davis, 2023). In particular, we recognize the importance of assessing strength and body composition, precisely criteria for sarcopenia such as handgrip strength, gait speed, and appendicular lean mass. These factors play a crucial role in understanding the effects of different training modalities, especially in women with varying blood pressure levels. By assessing these parameters, our study may need a comprehensive view of the potential influences of strength and body composition on the observed outcomes. Thus, future studies that can add these variables to this theme will bring strong evidence to highlight this theory. It is important to emphasize, at the end of this discussion, that existing literature already highlights the significance of physical activity and muscle strength on the quality of life. Physical activities play a pivotal role in maintaining overall health and vitality in older adults, and our data complement the existing findings in the literature (Smith and Johnson, 2020; Brown and Davis, 2023).

Considering all these findings, it is recommended that future studies be conducted to address the mentioned limitations and advance the knowledge about the effects of flexibility and multicomponent training. By doing so, more robust results can be obtained, contributing to the development of effective strategies for improving health and quality of life in different populations.

The idea of finding an interconnection between postural misalignment and blood pressure is new and this study is exploratory in nature. Many hypotheses generated from our results cannot be confirmed by this study, and further studies are needed to validate them, such as muscle tone as a possible modifier of PVR and evidence of sympathetic nerve activation caused by posture (Pollak et al., 1976). The assessment of muscle tone demands the use of tools not available at the time, such as electromyography and evaluation of sympathetic nervous activity. The association between postural misalignment and BP is significant as it is a new approach, opening perspectives for assessment and treatment.

## 5 Conclusion

Multicomponent training when associated with flexibility training presents better results for body alignment and adjustment in the COM. In addition, this improvement in angles such as hip, knee, lumbar, and head are associated with improved blood pressure. Flexibility training groups associated with multicomponent training demonstrated greater reduction in sBP and dBP values, while hypertensive individuals who performed only multicomponent training presented reductions only in systolic blood pressure. These data reveal the importance of flexibility training and improved adjustment in the postural pattern of hypertensive individuals.

## Data Availability

The raw data supporting the conclusion of this article will be made available by the authors, without undue reservation.
