# Supplementary material for: Fourteen weeks of multicomponent training associated with flexibility training modifies postural alignment, joint range of motion and modulates blood pressure in physically inactive older women: a randomized clinical trial
Source: Front Physiol. 2023 Nov 2;14:1172780. doi: 10.3389/fphys.2023.1172780 (PMC10664174; doi:10.3389/fphys.2023.1172780)
Supplement: Supplementary file 1 [file Table1.pdf]

Supplementary Material

Article Title

Andressa C. S. Sobrinho*, Cicero Jonas R. Benjamim, Mariana Luciano de Almeida, Guilherme da Silva Rodrigues, Laryssa Grazielle Feitosa Lopes, João Gabriel Ribeiro de Lima, Carlos Roberto Bueno Júnio

*** Correspondence:** Andessa Crystine da Silva Sobrinho andressa.sobrinho@usp.br

1 Supplementary Tables

Supplementary Table 1- Critical Values for Normality Test in Blood Pressure, Heart Rate, and Plane Asymmetry Variables.

Variables	Test							
	Anderson Darling		D'agostino & Person		Shapiro-Wilk		Kolmogorov-Smirnov	
	A2	P value	K2	P value	W	P value	KS	P value
**Heart rate pre (bpm)**	0.75	0.04	4.69	0.09	0.96	0.01	0.09	0.02
**Heart rate post (bpm)**	1.12	0.00	5.42	0.96	0.96	0.01	0.13	0.00
**sBP pre (mmHg)**	1.30	0.00	5.47	0.06	0.95	0.00	0.11	0.00
**sBP post (mmHg)**	1.02	0.01	9.89	0.00	0.96	0.00	0.10	0.00
**dBP pre (mmHg)**	1.50	0.00	17.91	0.00	0.95	0.00	0.10	0.00
**dBP post (mmHg)**	1.54	0.00	16.85	0.00	0.94	0.00	0.09	0.01
**fractional change (delta) sBP**	0.60	0.11	3.87	0.14	0.98	0.05	0.07	0.07
**fractional change (delta) dBP**	0.66	0.08	1.25	0.53	0.98	0.06	0.06	>0.10

**frontal plane asymmetry 1 (º)**	1.21	0.00	19.01	0.00	0.94	0.00	0.09	0.01
**frontal plane asymmetry 2 (º)**	1.20	0.00	7.25	0.02	0.96	0.00	0.12	0.00
**asymmetry in the sagittal plane 1 (º)**	2.06	0.00	3.47	0.17	0.94	0.00	0.13	0.00
**asymmetry in the sagittal plane 2 (º)**	2.36	0.00	8.46	0.01	0.94	0.00	0.13	0.00

Subtitle - * p<0.05

Supplementary Table 2 - Functional Flexibility Test

Variable	Group (n)	Pre	Post	F / p Value (group)	F / p Value (time)	F / p Value (group x time)
**Sit-and-reach (cm)**	FT (24)	-1.35±11.2	2.8±8.5*	1.695 / 0.140	6.007 / 0.016	6.831 / <0.001
	FTSAH (28)	1.6±9.3	5.3±8.7*			
	MT (22)	-4.9±8.1	2.5±3.4*			
	MTSAH (23)	0.3±4.9	0.6±6.6			
	CG (18)	1.2±11.6	-4.1±8.6[a,d]			
	CGSAH (26)	-1.0±8.2	-1.9±6.0			
**Hands-on-back (cm)**	FT (24)	-5.5±8.9	-2.1±6.6	2.050 / 0.076	0.011 / 0.915	2.309/ 0.047
	FTSAH (28)	-12.4±9.0	-6.6±8.2			
	MT (22)	-8.8±10.8	-7.2±11.3			
	MTSAH (23)	-9.5±10.0	-7.7±9.1			
	CG (18)	-6.6±10.2	-4.9±9.4			
	CGSAH (26)	-8.4±12.4	-8.6±11.7			

Subtitle – FT: flexibility training; FTSAH: flexibility training for hypertensive patients; MT: Multicomponent Training; MTSAH: multicomponent training for hypertensive patients; CG: control group; CGSAH: hypertensive control group; * p<0.05 for differences between pre and post groups (two-way ANOVA test was used for repeated measures, followed by Tukey Post-Hoc; a: FT difference at the same time point d: MTSAH difference at the same time point;

Supplementary Table 3 - Joint range of motion test, in flexion and extension movements

Variable	Group (n)	Pre	Post	F / p Value (group)	F / p Value (time)	F / p Value (group x time)
**CERVICAL Ext (º)**	FT (24)	16.0±9.5	19.5±8.9[*]	1.534 / 0.183	37.1 / <0.001	6.9 /< 0.001
	FTSAH (28)	13.4±6.9	15.4±6.9[*]			
	MT (22)	13.9±7.1	16.3±8.8[*]			
	MTSAH (23)	12.7±5.0	13.0±6.0			
	CG (18)	12.8±6.6	12.7±8.3			
	CGSAH (26)	12.4±6.4	12.6±7.3			
**CERVICAL Flex (º)**	FT (24)	35.2±14.3	39.0±14.5[*]	1.982 / 0.085	40.9 / <0.001	15.7 / <0.001
	FTSAH (28)	31.5±14.6	36.1±14.6[*]			
	MT (22)	28.7±14.8	32.0±16.7[*]			
	MTSAH (23)	36.0±15.4	36.2±15.7			
	CG (18)	35.9±10.3	35.0±11.0			
	CGSAH (26)	27.8±12.2	27.0±11.5[a,b,c,d]			
**SHOULDER Ext (º)**	FT (24)	29.0±10.7	31.5±10.6[*]	1.430 / 0.217	99.1 / <0.001	13.7 / <0.001
	FTSAH (28)	28.9±7.2	30.8±7.8[*]			
	MT (22)	26.3±9.3	28.8±9.2[*]			
	MTSAH (23)	32.9±8.8	33.6±7.4			
	CG (18)	27.6±7.5	27.6±8.2			
	CGSAH (26)	32.4±9.6	32.6±10.2			
**SHOULDER Flex (º)**	FT (24)	137.7 ±21.1	142.8±20.6[*]	4.375 / 0.001	24.7 / <0.001	5.8 / <0.001
	FTSAH (28)	135.3±19.4	140.5±19.8[*]			
	MT (22)	141.6±19.8	146.1±17.5[*]			
	MTSAH (23)	150.6±16.0	151.4±14.2[a,d]			
	CG (18)	154.2±24.0	152.9±24.6[a],[b,d,]			
	CGSAH (26)	158.2±18.5	158.4±19.1[a],[b,d]			
**LOW BACK Ext (º)**	FT (24)	17.5±10.4	22.7±10.4[*]	0.943 / 0.456	119.2 / <0.001	11.2 / <0.001
	FTSAH (28)	15.6±6.8	19.7±7.1[*]			
	MT (22)	16.1±9.0	20.0±10.7[*]			
	MTSAH (23)	16.1±7.8	16.8±8.4			
	CG (18)	17.0±10.4	18.3±11.7[a*]			

	CGSAH (26)	14.5±7.9	15.1±9.5			
**LOW BACK Flex (º)**	FT (24)	74.5±9.2	79.5±10.2[*]	0.583 / 0.713	79.7 / <0.001	15.7 / <0.001
	FTSAH (28)	75.5±9.0	79.0±9.9[*]			
	MT (22)	73.9±10.0	76.1±11.8[*]			
	MTSAH (23)	75.7±11.2	76.3±10.7			
	CG (18)	73.6±11.0	73.4±12.4[a,d]			
	CGSAH (26)	73.8±10.8	73.5±11.9			
**HIP Ext (º)**	FT (24)	5.0±2.6	7.6±2.6[*]	3.144 / 0.010	57.4 / <0.001	15.1 / <0.001
	FTSAH (28)	5.5±1.9	7.8±2.0[*]			
	MT (22)	5.4±1.8	8.1±3.7[*]			
	MTSAH (23)	4.6±1.6	4.8±3.8 [a,b]			
	CG (18)	5.5±1.0	5.4±2.0 [a], [b,d]			
	CGSAH (26)	5.5±1.8	4.9±3.0[a]			
**HIP Flex (º)**	FT (24)	62.2±9.6	71.7±9.6[*]	2.5 / 0.033	68.2 / <0.001	7.2 / <0.001
	FTSAH (28)	63.1±10.6	71.5±10.5[*]			
	MT (22)	54.5±14.9	60.5±16.5[*]			
	MTSAH (23)	59.8±17.6	66.4±18.0 [a,b]			
	CG (18)	53.1±17.9	54.6±21.6 [a,b,d]			
	CGSAH (26)	58.8±19.4	59.5±21.7 [c,e]			
**KNEE Ext (º)**	FT (24)	91.3±18.3	95.3±18.6[*]	1.980 / 0.085	0.06 / 0.807	4.9 / <0.001
	FTSAH (28)	93.8±19.7	97.6±19.8			
	MT (22)	93.6±18.8	95.5±20.5			
	MTSAH (23)	81.9±18.5	80.7±21.4[a,]			
	CG (18)	97.1±18.8	96.0±18.9[*]			
	CGSAH (26)	93.3±22.2	87.0±23.2[a, d,]			
**KNEE Flex (º)**	FT (24)	87.5±27.6	91.5±28.0[*]	0.790 / 0.559	0.12 / 0.720	6.9 / <0.001
	FTSAH (28)	94.5±25.5	98.2±25.6[a*]			
	MT (22)	90.1±15.3	92.0±16.8[d]			
	MTSAH (23)	85.5±20.6	84.3±22.7			
	CG (18)	97.1±18.8	92.7±19.4[*]			
	CGSAH (26)	93.3±22.2	90.4±21.6[d]			
**ANKLE Ext (º)**	FT (24)	10.3±3.3	14.0±3.6[*]	5.665/ <0.001	19.4 / <0.001	19.8 / <0.001

	FTSAH (28)	10.0±3.2	13.0±3.4*			
	MT (22)	8.8±3.6	10.1±5.5[a,d]			
	MTSAH (23)	9.6±3.5	9.6±4.9			
	CG (18)	9.4±3.3	7.5±4.0[a,b,d*]			
	CGSAH (26)	8.2±2.4	7.0±3.5[a,d,e]			
**ANKLE Flex (º)**	FT (24)	19±8.0	24.3±7.6*	0.901 / 0.482	46.1 / <0.001	4.6 / 0.001
	FTSAH (28)	17.1±7.7	20.8±8.0*			
	MT (22)	20.8±7.3	23.3±6.9*			
	MTSAH (23)	17.8±8.0	19.7±8.2			
	CG (18)	19.7±7.5	20.2±8.4[a]			
	CGSAH (26)	19.1±8.0	19.6±8.3			

Subtitle – FT: flexibility training; FTSAH: flexibility training for hypertensive patients; MT: Multicomponent Training; MTSAH: multicomponent training for hypertensive patients; CG: control group; CGSAH: hypertensive control group Ext: Extension; Flex: Flexion; * $p<0.05$ for differences between pre and post groups (two-way ANOVA test was used for repeated measures, followed by Tukey Post-Hoc; a: FT difference at the same time; b: FTSAH difference at the same time; c: MT difference at the same time; d: MTSAH difference at the same time; e: CG difference at the same time; f: CGSAH difference at the same time

Supplementary Table 4 - Assessment of Plane Asymmetry Variables in the Frontal and Sagittal Planes.

Variable	Group (n)	Pre	Post	F / p Value (group)	F / p Value (time)	F / p Value (group x time)
**Asymmetry in the Frontal Plane (º)**	FT (24)	54.3±22.0	33.0±19.8*	3.384 / 0.007	35.0 / <0.001	6.1 / <0.001
	FTSAH (28)	54.3±19.1	34.2±14.7*			
	MT (22)	45.1±18.6	43.8±18.8			
	MTSAH (23)	48.9±15.9	36.4±15.3*			
	CG (18)	60.5±27.3	59.3±21.9[a,b,d]			
	CGSAH (26)	54.2±23.6	54.12±23.0[a,b,d,e]			
**Asymmetry in the Sagittal Plane (º)**	FT (24)	34.8±9.9	24.5±10.9*	5.664/ <0.001	28.1 / <0.001	4.6 / 0.001
	FTSAH (28)	34.1±10.1	23.7±10.4*			
	MT (22)	36.7±9.9	29.0±11.6[d*]			
	MTSAH (23)	37.2±9.8	26.0±9.2*			
	CG (18)	36.0±17.0	39.0±9.8[a,b,d]			
	CGSAH (26)	39.4±14.2	39.2±8.4[a,b,d,e]			

Subtitle - FT: flexibility training; FTSAH: flexibility training for hypertensive patients; MT: Multicomponent Training; MTSAH: multicomponent training for hypertensive patients; CG: control group; CGSAH: hypertensive control group; * p<0.05 for differences between pre and post groups (two-way ANOVA test was used for repeated measures, followed by Tukey Post-Hoc; a: FT difference at the same time; b: FTSAH difference at the same time; c: MT difference at the same time; d: MTSAH difference at the same time; e: CG difference at the same time; f: CGSAH difference at the same time
